# Anxiety and Well-Being: A Factorial and Regression Analysis of Life Satisfaction Determinants in a Japanese Population

**DOI:** 10.31662/jmaj.2025-0383

**Published:** 2026-02-06

**Authors:** Haruka Ishii, Yoko Ishii

**Affiliations:** 1 Academic Assembly Faculty of Medicine, University of Toyama, Toyama, Japan; 2 Graduate School of Health and Nutrition Sciences, The University of Nagano, Nagano, Japan

**Keywords:** subjective well-being, life satisfaction, anxiety-related factors

## Introduction

Self-reports of psychological well-being have become a subject of intense debate in public policy and economics, with the improvement of population well-being emerging as a key societal goal. In its well-being survey *How’s Life 2020?*, the Organization for Economic Co-operation and Development (OECD) reported that Japanese respondents rated their overall life satisfaction at an average of 6.1 on a scale of 0 to 10―lower than the OECD average of 6.7 ^(1)^.

Previous frameworks such as the OECD Better Life Index and the Social Production Function theory have identified income and jobs, health and life satisfaction, and environmental quality as key components of well-being ^(2), (3)^.

Diener et al. ^(4)^ proposed theories suggesting that various components of subjective well-being―such as life satisfaction, positive affect, and negative affect―are influenced by different factors. A study on perceptions of past, present, and future life satisfaction among adults aged 33-79 years found that Japanese adults began to expect declines in life satisfaction at an earlier age than their United States counterparts ^(5)^.

Nishimura and Yagi conducted a factor analysis of the Oxford Happiness Questionnaire ^(6)^, a widely used measure of psychological well-being, to identify factors related to positive ideation and anxiety. In a sample of 20,000 Japanese individuals, they found that the negative correlation between anxiety and subjective well-being was stronger than the positive correlation with positive ideation ^(7)^. This suggests that anxiety-related factors have a more substantial impact on the subjective well-being of Japanese people than positive factors associated with satisfaction, contributing to lower overall life satisfaction.

Anxious temperament has also been identified as a significant risk factor for suicide-related ideation in the Japanese adult population ^(8)^. Despite its importance, research on anxiety-related determinants of subjective well-being in Japan remains limited ^(5), (9)^. In light of the limited empirical evidence, the present study employs factor analysis to identify latent psychosocial dimensions―including anxiety-related factors―and examines their relative contributions to life satisfaction in the Japanese population. By incorporating anxiety-related variables into a multidimensional framework, we aim to provide a more comprehensive understanding of the mechanisms underlying life satisfaction and to inform future research and policy development.

## Materials and Methods

Data were obtained from the *2022 Survey on Satisfaction and Quality of Life*. This survey was deposited by the Cabinet Office of Japan, conducted by Survey Research Center Co., Ltd. in February 2022, and provided through the Social Science Japan Data Archive, Center for Social Research and Data Archives, Institute of Social Science, The University of Tokyo. The target population consisted of internet panel registrants residing in Japan, aged between 15 and 89 years. Sampling was based on prefecture, gender, and age group composition ratios. A total of 10,631 respondents were included. Questions on life satisfaction and related factors were drawn from the survey instrument.

Life satisfaction was assessed with the question: *“How satisfied are you with your current life overall?”* Responses were given on a scale from 0 (“Not satisfied at all”) to 10 (“Very satisfied”).

Anxiety-related factors were assessed with the question: *“When thinking about the future, how would you rate your level of anxiety in various aspects of life?”* Responses were given on a scale from 0 (“Very anxious”) to 10 (“Not anxious at all”) for 13 categories For statistical processing, scores were converted from 0-10 to 1-11 for ease of calculation.

The Shapiro-Wilk test indicated that life satisfaction scores were not normally distributed. Therefore, non-parametric tests were applied: the Mann-Whitney U test for two-group comparisons and the Kruskal-Wallis test for three or more groups, with adjusted significance levels for pairwise comparisons. Inter-factor correlations were assessed using Spearman’s rank correlation coefficient. Internal consistency was evaluated using Cronbach’s alpha. Factor analysis was performed using the principal factor method with Promax rotation, and multiple regression analysis was conducted with extracted factors as independent variables.

All analyses were performed using SPSS version 28.0 (IBM Japan, Ltd., Tokyo, Japan), with a significance level of 0.05.

Ethics approval was not required because this study involved secondary analysis of publicly available data from the University of Tokyo’s Center for Social Research and Data Archives, Institute of Social Science. The microdata was used in full compliance with the pledge statement, ensuring adherence to all stipulated conditions regarding confidentiality, academic use, and data handling.

## Results

Table 1 summarizes participants’ demographic characteristics.

**Table 1.** The Demographic Characteristics of the Participants.

		Number	%			
The study participants	Total	10631				
	Men	5296	49.8			
	Women	5335	50.2			
						
		Mean	S.D.	Median	Minimum	Maximum
Age	Total	42.78	16.77	41	15	89
	Men	42.80	16.84	41	15	88
	Women	42.76	16.81	41	15	89
						
		Number	%			
Age	15-19	323	3.0			
	20-29	2627	24.7			
	30-39	2133	20.1			
	40-49	1896	17.8			
	50-59	1345	12.7			
	60-69	1557	14.6			
	70-79	688	6.5			
	80-89	62	0.6			
						
			Number	%		
Age group	Men	15-39	2506	23.6		
		40-59	1655	15.6		
		60-89	1135	10.7		
	Women	15-39	2577	24.2		
		40-59	1586	14.9		
		60-89	1172	11.0		
						
Number of household members (including oneself)	Men	1 (living alone)	1136	10.7		
		2	1283	12.1		
		3	1281	12.0		
		4	1031	9.7		
		5 or more	565	5.3		
	Women	1 (living alone)	960	9.0		
		2	1602	15.1		
		3	1277	12.0		
		4	997	9.4		
		5 or more	499	4.7		
						
Highest education	Junior high school		270	2.5		
	High school		2981	28.0		
	Vocational school		1251	11.8		
	2 or 3 years college		1134	10.7		
	University		4483	42.2		
	Graduate school		512	4.8		
						
Annual household income	less than 1 million yen					
	1~3 million yen		723	6.8		
	3~5 million yen		4784	45.0		
	5~10 million yen		4003	37.7		
	10~20 million yen		941	8.9		
	20~50 million yen		136	1.3		
	50~100 million yen		12	0.1		
	100 million yen or more		32	0.3		
						
		Number	%		
Residential prefecture	Hokkaido	247	2.32		
	Aomori	215	2.02		
	Iwate	215	2.02		
	Miyagi	226	2.13		
	Akita	213	2.00		
	Yamagata	213	2.00		
	Fukushima	218	2.05		
	Ibaraki	228	2.14		
	Tochigi	219	2.06		
	Gunma	219	2.06		
	Saitama	264	2.48		
	Chiba	254	2.39		
	Tokyo	316	2.97		
	Kanagawa	279	2.62		
	Nigata	223	2.10		
	Toyama	212	1.99		
	Ishikawa	214	2.01		
	Fukui	212	1.99		
	Yamanashi	212	1.99		
	Nagano	220	2.07		
	Gifu	219	2.06		
	Shizuoka	235	2.21		
	Aichi	264	2.48		
	Mie	217	2.04		
	Shiga	215	2.02		
	Kyoto	227	2.14		
	Osaka	276	2.60		
	Hyogo	249	2.34		
	Nara	215	2.02		
	Wakayama	212	1.99		
	Tottori	211	1.98		
	Shimane	212	1.99		
	Okayama	219	2.06		
	Hiroshima	227	2.14		
	Yamaguchi	215	2.02		
	Tokushima	212	1.99		
	Kagawa	212	1.99		
	Ehime	216	2.03		
	Kochi	212	1.99		
	Fukuoka	245	2.30		
	Saga	212	1.99		
	Nagasaki	216	2.03		
	Kumamoto	217	2.04		
	Ohoita	214	2.01		
	Miyazaki	213	2.00		
	Kagoshima	216	2.03		
	Okinawa	214	2.01		

**Figure 1.**
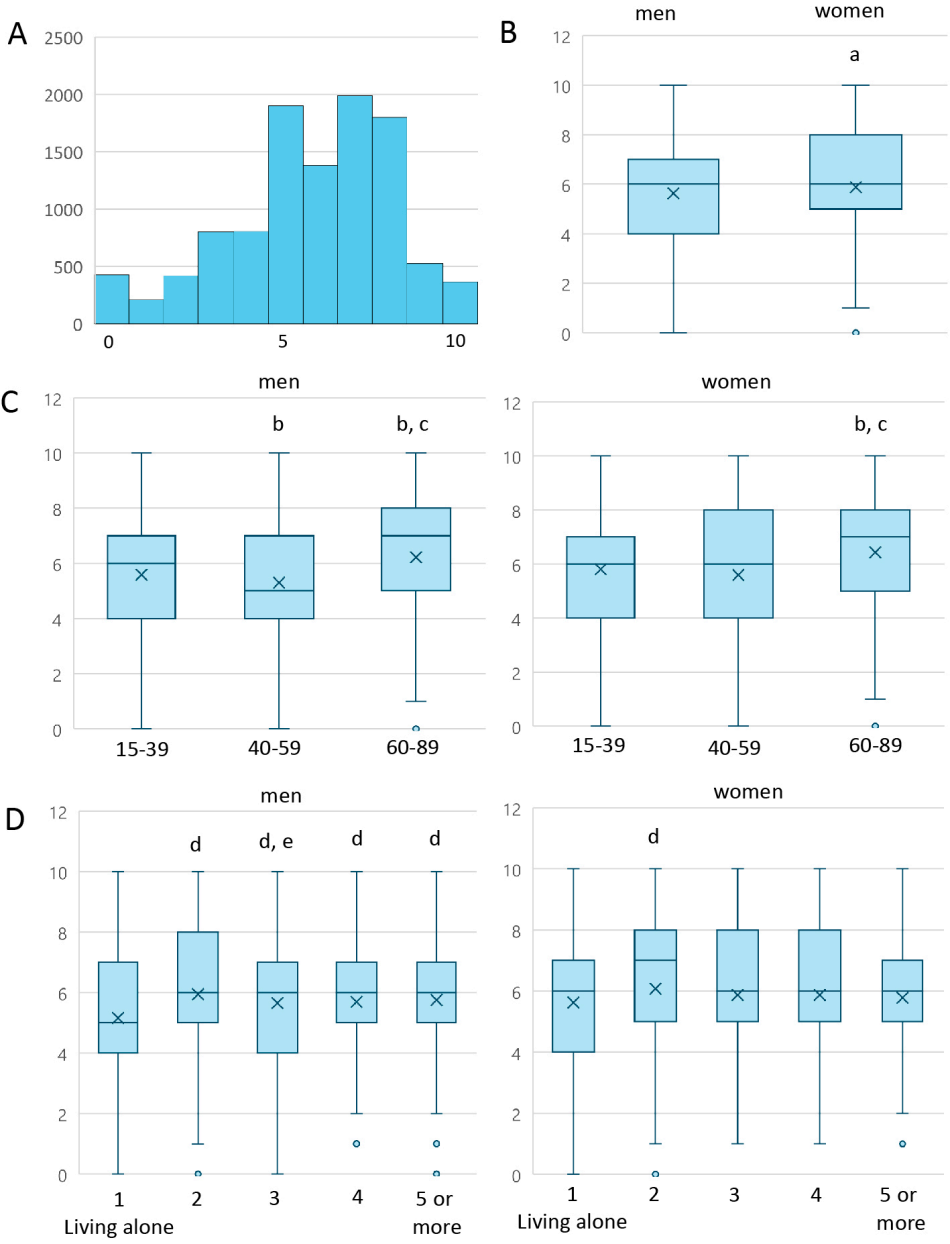
Histograms of life satisfaction scores (A) and mean and median scores by gender (B), age group (C), and household size (D). (A) Life satisfaction was assessed with the question: *“How satisfied are you with your current life overall?”* Responses were given on a scale from 0 to 10, where 0 means “Not satisfied at all” and 10 means “Very satisfied.” (B-D) In the boxplots, median values are represented by a horizontal line and mean values by a cross mark. The y-axis extends to 12 to accommodate the confidence intervals; however, life satisfaction is rated on a scale from 0 to 10. (B) a: p < 0.001 versus men (Mann-Whitney U test). (C, D) b: p < 0.001 versus younger group (15-39 years); c: p < 0.001 versus middle-aged group (40-59 years); d: p < 0.001 versus single-person households; e: p < 0.01 versus two-person households; f: p < 0.05 versus two-person households (Kruskal-Wallis test).

Figure 1A shows the distribution of life satisfaction scores (mean = 5.76 ± 2.35; median = 6). Figure 1B indicates a significant gender difference (men: 5.63 ± 2.40; women: 5.88 ± 2.30; median = 6 for both; p < 0.001, Mann-Whitney U test). Figures 1C and D present mean and median scores by age group and household size.

Kruskal-Wallis tests revealed significant age-related differences for men across all comparisons (15-39 vs. 40-59 years: p = 0.001; 40-59 vs. 60+: p < 0.001; 15-39 vs. 60+: p < 0.001). For women, differences were significant between 60+ years and both younger groups (p < 0.001), but not between 15-39 and 40-59 years (p = 0.413).

Life satisfaction by household size was lowest for single-person households and highest for two-person households. Among men, most pairwise comparisons were significant (e.g., 1 vs. 2 persons: p < 0.001; 2 vs. 3 persons: p = 0.005). Among women, significant differences were mainly between one- and two-person households (p < 0.001) and between two- and five-or-more-person households (p = 0.040).

Spearman’s rank correlations showed all 13 anxiety factors were moderately and positively associated with life satisfaction (ρ = 0.44-0.62; p < 0.001; Table 2A). Cronbach’s alpha was 0.95, indicating high internal consistency. Variance Inflation Factor (VIF) values (1.99-3.84) suggested no multicollinearity.

**Table 2A.** Associations between 13 Anxiety-Related Factors and Current Life. Satisfaction: Spearman’s Rank Correlations.

“When thinking about the future, how anxious do you feel about various aspects of life (13 items)? If 0 represents ‘very anxious’ and 10 represents ‘not anxious at all’, which number best reflects your level of anxiety? Please choose only one number.”	Spearman’s rho for Life Satisfaction	Cronbach’s alpha coefficient
Household finances and assets	0.620***	0.95
Employment environment and wages	0.559***	
Housing conditions	0.573***	
Work-life balance	0.595***	
Personal health status	0.548***	
Educational level and environment	0.558***	
Social connections, such as friendships and community involvement	0.558***	
Trust in political institutions, public administration, and the judiciary	0.403***	
Natural environment such as air and water	0.472***	
Personal safety	0.495***	
Ease of child-rearing	0.459***	
Ease of caregiving (giving and receiving)	0.443***	
Enjoyment and interest in life	0.681***	

Anxiety-related factors were assessed using the question: *When thinking about the future, how would you rate your level of anxiety in various aspects of life?* Responses were given on a scale from 0 to 10, where 0 means “Very anxious” and 10 means “Not anxious at all.” Participants selected one number for each of the 13 categories. Spearman’s rho, ***p < 0.001.

Exploratory factor analysis (principal factor method, Promax rotation) extracted three factors explaining 66.74% of variance: Quality of Living Environment (α = 0.916), Personal Quality of Life (α = 0.874), and Economic Stability (α = 0.725). Inter-factor correlations were positive (0.67-0.78; Table 2B). To assess the suitability for factor analysis, the Kaiser-Meyer-Olkin (KMO) measure and Bartlett’s test of sphericity were conducted. The KMO value was .957, indicating excellent sampling adequacy. Bartlett’s test of sphericity was significant, χ^2^ (78) = 105,728.07, p < .001, suggesting sufficient correlations among variables. Therefore, factor analysis was deemed appropriate.

**Table 2B.** Associations between 13 Anxiety-Related Factors and Current Life. Satisfaction: Exploratory Factor Analysis.

		Factors			The three extracted factors	Cronbach’s alpha coefficient
		1	2	3		
The level of future anxiety about	Enjoyment and interest in life	0.733	0.055	0.095	Personal Quality of Life	0.916
	Social connections, such as friendships and community involvement	0.639	0.134	0.063		
	Personal health status	0.622	0.100	0.088		
	Educational level and environment	0.616	0.123	0.093		
	Work-life balance	0.516	0.002	0.390		
	Housing conditions	0.514	0.122	0.212		
	Natural environment such as air and water	0.251	0.733	-0.142	Quality of Living Environment	0.874
	Personal safety	0.286	0.713	-0.129		
	Ease of child-rearing	0.040	0.597	0.183		
	Ease of caregiving (giving and receiving)	-0.072	0.581	0.341		
	Trust in political institutions, public administration, and the judiciary	-0.113	0.551	0.334		
	Employment environment and wages	0.100	-0.009	0.836	Economic Stability	0.725
	Household finances and assets	0.239	0.010	0.663		
						
Factor correlation matrix		1	2	3		
		1.000	0.776	0.746		
			1.000	0.676		
				1.000		

An exploratory factor analysis was conducted on the 13 factors using the principal factor method with Promax rotation. The final factor pattern and inter-factor correlations after Promax rotation resulted in three extracted factors.

Multiple regression analysis (Table 2C) showed Personal Quality of Life had the strongest effect on life satisfaction (β = 0.68, p < 0.001), followed by Economic Stability (β = 0.21, p < 0.001). Quality of Living Environment was significant but weaker (β = 0.097, p < 0.001). The model fit was good (Adjusted R^2^ = 0.608), and ANOVA confirmed overall significance (F = 2359.532, p < 0.001).

**Table 2C.** Associations between 13 Anxiety-Related Factors and Current Life. Satisfaction: Multiple Regression Analysis.

		B	SE	β	p	VIF
Anxiety factors	Personal quality of life	0.895	0.013	0.678	< 0.001	1.068
	Quality of living environment	-0.136	0.014	-0.097	< 0.001	1.125
	Economic stability	0.224	0.011	0.207	< 0.001	1.035
Control variable	Household size	0.000	0.000	-0.013	0.030	1.034
	Highest education	-0.020	0.010	-0.012	0.058	1.050
	Annual household income	0.051	0.010	0.032	< 0.001	2.809
	Age group	0.000	0.000	0.032	< 0.001	2.604
	Gender	0.200	0.029	0.042	< 0.001	2.666

Multiple regression analysis was performed to examine the influence on life satisfaction. The dependent variable was life satisfaction, and the independent variables were the three factors extracted from the factor analysis. Gender, age group, household size, total annual household income, and highest education level were included as covariates. B: unstandardized coefficient; β: standardized coefficient; SE: standard error.

## Discussion

Life satisfaction varied significantly by gender, age, and household size. The overall mean score (6.76; median = 7) indicates a moderately high level of satisfaction. Women reported slightly higher scores than men, consistent with previous findings on gender differences in well-being ^(10)^. Age-related differences were more pronounced among men, with all pairwise comparisons reaching significance. Among women, only comparisons involving the oldest age group (60+) were significant, aligning with prior research on aging and well-being ^(11)^.

Global surveys, such as the Gallup World Poll, have documented a U-shaped relationship between well-being and age, with the lowest levels around midlife ^(12)^.

Household size also influenced life satisfaction: individuals living alone reported the lowest scores, while those in two-person households reported the highest. These differences were more pronounced among men, whereas women exhibited fewer significant contrasts. This trend aligns with the findings of Hori and Kamo, who reported that marital status and household composition are more strongly associated with happiness among men, while women’s well-being is less dependent on these factors ^(13)^.

An analysis of 13 psychosocial and environmental factors revealed moderate positive correlations with life satisfaction, suggesting that multiple domains contribute to overall well-being. Factor analysis identified three key dimensions―Quality of Living Environment, Personal Quality of Life, and Economic Stability―which together explained 66.74% of the variance. High internal consistency and positive inter-factor correlations indicate that these domains are both reliable and interrelated.

Regression analysis confirmed Personal Quality of Life as the strongest predictor (β = 0.68), followed by Economic Stability (β = 0.21) and Quality of Living Environment (β = 0.097).

These findings align with the Social Production Function framework, which highlights personal experiences and financial security as central drivers of life satisfaction ^(3)^. Furthermore, cross-national data from the OECD Better Life Index suggest that while environmental quality contributes to well-being, its impact is often mediated by economic and social conditions―particularly in countries with lower income levels or less stable infrastructure ^(2)^. Swami et al. ^(14)^ similarly found that across 65 nations, financial security and relationship status were more strongly associated with life satisfaction than environmental factors, indicating a potentially universal pattern. The relatively smaller effect of environmental quality may reflect its indirect influence through personal and economic domains.

These findings also have important cultural and policy implications. For example, the Cabinet Office conducts the *Survey on Satisfaction and Quality of Life* to monitor subjective well-being and related indicators ^(15)^. Our multidimensional approach aligns with the Quality of Life framework adopted in policy evaluation and complements SDGs-related goals on health and social inclusion ^(16)^. By identifying psychosocial dimensions that influence life satisfaction, this study offers preliminary insights that may contribute to the development of targeted interventions and inform policy considerations aimed at enhancing population well-being in Japan.

Overall, these results underscore the importance of a holistic approach that integrates demographic, personal, and environmental factors. Future research should explore how these domains interact over time and across diverse populations to inform targeted interventions and policy development.

### Limitations

This study used an online sample, which may limit generalizability, though regional distribution approximated national demographics. The cross-sectional design precludes causal inference; longitudinal studies are needed. Additionally, not all relevant factors could be assessed.

### Conclusion

Life satisfaction is influenced by demographic and psychosocial factors. Older adults and individuals in two-person households reported higher well-being. Among the predictors, concerns about Personal Quality of Life and Economic Stability had the strongest impact, while environmental quality played a smaller but meaningful role. These findings suggest that effective interventions should address both structural and personal vulnerabilities, and future research should explore how these factors interact across different life stages.

## Article Information

### Acknowledgments

The data for this secondary analysis, “*2022 Survey on Satisfaction and Quality of Life*,” deposited by the Cabinet Office of Japan, was provided by the Social Science Japan Data Archive, Center for Social Research and Data Archives, Institute of Social Science, The University of Tokyo.

### Author Contributions

Haruka Ishii: data analysis and manuscript writing; Yoko Ishii: project development, data management, and manuscript writing/editing.

### Conflicts of Interest

None
